# ‘It kinda sucks’: Illness perception of a group of South African adolescents with type 1 diabetes mellitus

**DOI:** 10.4102/phcfm.v13i1.2782

**Published:** 2021-02-17

**Authors:** Schvaugn Lesage, Elmarí Deacon, Esmé van Rensburg, David Segal

**Affiliations:** 1 Optentia Research Focus Area, Faculty of Health Sciences, North West University, Potchefstroom, South Africa; 2 COMPRES Research Focus Area, Faculty of Health Sciences, North West University, Potchefstroom, South Africa

**Keywords:** illness perception, adolescents, diabetes management, poor glycaemic control, thematic analysis

## Abstract

**Background:**

Living with diabetes is challenging, especially for adolescents at risk of poor glycaemic control. Understanding the illness perceptions of this group is important to be able to develop interventions for this growing population in need.

**Aim:**

This study explored the illness perception amongst adolescents living with type 1 diabetes (T1D) and how these perceptions interacted with the management of T1D.

**Setting:**

This study was conducted at a medical centre providing care for adolescents living with T1D in Parktown, South Africa.

**Methods:**

A qualitative, explorative design with semi-structured interviews was followed. A non-random purposive sampling method was utilised. The illness perception amongst eight adolescents, aged 12–18 years, at risk of poor glycaemic control, was analysed through thematic analysis.

**Results:**

Two subthemes related to illness perception were generated, namely (1) illness perception of T1D is negative and (2) living with T1D leads to a sense of being different. Furthermore, two subthemes were generated in relation to how illness perceptions interacted with diabetes management, namely (3) management of T1D is challenging and (4) management of T1D is motivated by fear.

**Conclusion:**

This group of adolescents with at-risk glycaemic control believed that T1D is difficult to manage, leading to a largely negative perception of the disease. This study contributes to the body of literature on adolescents where illness perception may play a role in adhering to diabetes care plans. This research may give additional insights into the awareness of illness perception in designing successful interventions.

## Introduction

The detrimental effect of diabetes in adolescents has been well researched, yet the prevalence of diabetes as a chronic condition amongst children and adolescents is increasing.^1^ Diagnosis of type 1 diabetes (T1D) peaks in adolescence^2^ with over 1.1 million children under the age of 20 living with T1D worldwide^3^ and the second most prevalent chronic condition in adolescences^4^ the age of 20 living with T1D worldwide (3) and the second most prevalent chronic condition in adolescences.^3^ Kalweit et al.^5^ indicated that the prevalence of diabetes in children aged 0–14 years in South Africa is currently approximately 0.8/100 000 individuals and increasing. However, according to Nuche-Berenguer et al.,^6^ awareness of the burdensome effect of a disease such as diabetes in the sub-Saharan context is relatively low, and it is often undiagnosed, leading to a short life expectancy. When T1D is not well managed, it can lead to a variety of complications, including microvascular disease, cardiovascular disease, blindness, amputations and even death.^2,3,7^ Poor management has also been associated with psychological disorders such as depression^6^ and anxiety.^8,9^

In Africa, poverty, lack of nutrition and inaccessible health services often hinder the necessary identification or management of diabetes. In their meta-analysis of literature, Nuche-Berenguer et al.^6^ found that in sub-Saharan countries, there are still a lack of diagnostic capabilities, inadequate access to medicine and poor capacity of healthcare workers. Basic services such as test strips for glucose meters are often not supplied by state hospitals in South Africa, especially in poorer or rural communities.^5^

Additionally to the type of health care support received,^10^ further identified barriers that prevent adherence to diabetes care plans amongst adolescents with T1D include lack of parental support,^10,11^ demographic factors such as race and age,^3^ attitudes,^2^ fatigue and peer pressure,^4^ coping styles^12^ and illness perception.^13,14^

Illness perception can be seen as a patient’s cognitive appraisal and belief system about his or her condition.^13^ Once it has been integrated with existing schemata to enable sense-making of symptoms,^15^ this guides his or her management of the condition.^16^ Illness perception has been used to predict physiological and behavioural outcomes in self-management,^17^ such as adherence to medication regimes.^18^ According to Broadbent et al.,^13^ the management of a variety of conditions can be improved by changing people’s perception of and attitudes towards their illness.

During adolescence, greater ownership and responsibility for diabetes management^19^ are crucial. During this phase, the adolescents’ perception, beliefs and habits regarding diabetes are formed^20^ as they attempt to make sense of their condition.^16^ However, these perceptions can change as adolescents gain greater cognitive and socio-emotional insight into their condition.^21^ Cosma et al.^22^ found that long-term beliefs held by adolescents about T1D predicted their emotional responses, which in turn may affect their behaviour. Schur et al.^23^ emphasise that by understanding an adolescent’s illness perception, the support provided can be more effective. Thus, illness perception plays a key role in the management of T1D. Limited research, mostly quantitative in nature, has been conducted around the illness perception of T1D^14,18,22,24^; however, a lacuna remains in research on the qualitative illness perception of adolescents with diabetes in the South African context. Exploring the illness perception amongst adolescents at risk of poor glycaemic control in South Africa will lead to improved understanding of T1D and thus aid in the management of diabetes in this age group.

It is worth noting that the definition of *well-controlled diabetes* differs.^2,25^ Whilst some literature refers to an HbA1C refers to glycated haemoglobin (A1C) (HbA1c) level of below 7.5% as optimal, the American Diabetes Association indicates that for adolescents, HbA1c levels of 7.5% – 10.0% can still be seen as fair and above or equal to 10.0% as poor. Criteria for well-controlled versus uncontrolled are more flexible for adolescents than for adults. This is because of the danger of hypoglycaemia and the fact that they are still developing physically whilst in the process of acquiring important diabetes management skills. The American Association Guidelines propose that for adolescents (13–18 years), good control can be seen as an HbA1c of less than 7.5%, fair control as 7.5% – 10.0% and poor control as achieving an HbA1C of above 10.0%.^5^ In this study, an HbA1c of greater than 7.5% was seen as at-risk glycaemic control.

Therefore, this study aimed to explore the illness perception amongst a group of South African adolescents with at-risk glycaemic control and how their illness perception may play a role in diabetes management.

## Methods

### Design

This study is explorative in nature. A qualitative research approach was utilised. The study aims to understand an individual’s experiences and perception,^26^ whilst the approach further allows in-depth study of phenomena.^27^ Qualitative research is holistic, allowing for the phenomena examined to be explored as they unfold in the real world as interrelated wholes.^28^ Therefore, this approach does not focus on the generalisability of the findings but rather the extent to which the results are transferable to other settings.^29^ Thus, this approach is well suited to gaining an in-depth understanding of the illness perception of adolescents with a risk of poor glycaemic control.

### Setting, population and sampling

The Centre for Diabetes and Endocrinology (henceforth CDE) in Parktown, South Africa served as the gatekeeper who informed prospective participants about the background to the study and the voluntary nature of participation. Non-random, purposive sampling was utilised to select participants who conformed to the sampling criteria. Eligibility criteria included (1) age between 12 and 18; (2) a diagnosis of T1D for at least 12 months prior to enrolment, thus to avoid any impact of continuous adjustments in treatment on the trustworthiness of data obtained;^30^ (3) an HbA1c level above 7.5% at the time of enrolment; (4) being a CDE patient so as to minimise treatment variables; (5) English- or Afrikaans speaking. Ineligible criteria included (1) suffering from another chronic medical condition that could impact diabetes management; (2) undergoing psychotherapy at the time of the research, as the psychotherapeutic process may alter their illness perception and as a precautionary measure to avoid interfering with the therapy process.

Prospective participants who were interested in taking part were screened to ensure eligibility. No participants dropped out after screening. Written informed consent was gathered from the adolescents and parental permission from their legal guardian. Data were collected from May 2018 to March 2019. The final sample consisted of eight adolescents whose biographical information and pseudonyms are expanded on in Table 1.

**TABLE 1:** Biographical information and pseudonyms of participants.

Participant’s pseudonym	Age	Gender	Age when diagnosed with T1D
Bill	13	M	3
John	14	M	11
Jane	13	F	5
Alice	13	F	5
Sue	13	F	10
Frank	14	M	10
Joy	16	F	9
Claire	12	F	9

T1D, type 1 diabetes; M, male; F, female.

The biographic distribution was as follows: female 63%, male 37%; the mean age was 13.5 years (range 12–16); the mean age of diagnosis was 8 (range 3–11); and the mean HbA1c level was 10.5% (range 8% – 14%).

### Data generation

Data were collected through a semi-structured interview schedule. An agenda was utilised to ensure consistency between the interviews. Additional open-ended probing questions were asked to obtain a deeper understanding of the materials. Six interviews were conducted at the CDE, in each case after the participants’ routine quarterly endocrinology appointment at this institution. Two participants requested to be interviewed at their homes in Gauteng, South Africa. The interview procedure took on an average 45 min. The interviews were audio recorded with consent from the participants and parental permission from their legal guardians.

### Data analysis and interpretation techniques

The first author transcribed the audio recordings verbatim, and each transcript was checked against the recording to ensure accuracy. Thematic analysis, as described by Braun et al.,^31,32^ was utilised, as it allows for the collection of rich and detailed data by identifying, analysing and reporting themes from within the data. This is achieved by minimising, organising and describing the data in detail. A recursive six-phase process characterises this kind of analysis, as follows: familiarisation of the data, initial code generation, theme searching, theme reviewing, defining and naming of themes and report writing. To ensure accuracy and reliability during data analysis and interpretation, the raw data were co-coded by the other researchers. No significant discrepancies were found. No new contributing themes emerged after interviewing five participants; that is, data saturation had occurred.

### Trustworthiness

To ensure the trustworthiness of the data, the study utilised the four criteria asserted by Lincoln et al.,^33^ namely credibility, transferability, dependability and confirmability. Credibility was attained through peer debriefing, that is informal discussions with a research colleague to uncover and examine potential biases and test hypotheses as well as member checking, that is allowing participants to review the preliminary findings. A thick description of the methodology and research process was provided to enhance transferability. Despite this, transferability might be limited because of the small sample size and lack of statistical analysis.^34^ However, Denscombe^35^ argues that despite the uniqueness of each case, it remains an example of a larger group, allowing for the possibility of transferability. Dependability was achieved through the co-coding process and by taking comprehensive field notes. Lastly, confirmability was achieved through researchers’ reflexivity and triangulation. In its turn, triangulation was achieved by collecting data from observations, audio recordings of the interviews and literature reviews that refined the understanding of the themes.

### Ethical considerations

This study was granted ethical approval by the North-West University’s Humanities and Health Research Ethics Committee (HHREC) of the Faculty of Humanities (NWU-HS-2017-0167). As the participants were under the age of 18 and had been diagnosed with T1D, the study was considered to be of medium ethical risk. Therefore, the researchers remained cognisant of the physical- and potentially psychological vulnerabilities of the participants throughout the study. To avoid participant stress, interviews were conducted in a familiar space, a low-carb snack was provided and free psychological services were offered if needed. The rights of the participants were upheld by ensuring confidentiality and voluntary participation. Anonymity was ensured by using participant codes and storing consent forms separately from the participant code list. Interviews commenced after independent, written, informed consent was received from all participants and parental permission was received from their legal guardians. The interviews were conducted in English, audio recorded and transcribed verbatim. Participants received a summary of the findings.

## Results

This study aimed to explore the illness perception amongst adolescents at risk of poor glycaemic control and how this perception may contribute to their management of the illness. The results were analysed in terms of illness perception, which yielded two subthemes, namely (1) perception of T1D is negative and (2) living with T1D leads to a sense of being different. In terms of their management thereof and how this may relate to illness perception, two subthemes came to the fore, namely (3) management of T1D is challenging and (4) management of T1D is motivated by fear. These themes are illustrated here in this article by appropriate verbatim extracts from the eight participants.

### Illness perception

The superordinate theme of illness perception yielded two subthemes: (1) feelings of negativity and (2) of being different.

#### Subtheme 1: Perception of type 1 diabetes is negative

Participants expressed negative feelings in response to T1D. When they thought of food constraints that it caused, they felt dejected and different from others, as they were unable to participate in social activities, which often centred on food matters such as those encountered at birthday parties and family reunions. These negative feelings led to a perception of T1D as negative. As a result, they preferred to avoid thinking about it.

The negative perception of T1D often resulted in feelings of sadness, which became clear when they related experiences of living with T1D. When asked how he experiences T1D, Bill (male, age 13) reported:

‘“Um, it kinda sucks …”. Claire said that it made her “sad,” expanding on this to say “only when I think about it and when there is cake is around.”’ (female, age 12)

Participants also felt that their T1D was holding them back in school, because they had to miss out on activities because of being ill. Jane indicated the following:

‘“… well sometimes I get a little held back in group activities … So sometimes that’s quite frustrating …” And Frank said: “… and sometimes it delays me and I get into trouble for that.”’ (female, age 13)

It is evident that adolescents with T1D face considerable emotional challenges of a great variety not shared by their peers.

The participants expressed that they tried not to think about T1D, as indicated above, because it evoked feelings of sadness and fear. Joy (female, age 16) expressed her feelings around this: ‘It is quite scary. I’m not used to focussing and talking about it, so yeah it’s quite scary.’ This tendency towards avoidance was also illustrated by one of Sue utterances:

‘Well, I don’t think too much because then I feel like it’s okay, I’ve accepted that I have, I have diabetes. I just have to move on and take care of myself.’ (female, age 13)

This tendency towards denial or avoidance included their understanding of T1D, because they did not think about what T1D meant beyond a medical definition. Alice said:

‘I actually really don’t know because like when I got it my parents just like told me and I was like ok. So, I guess I’ve just adapted to the word but never really thought about it.’ (female, age 13)

The intrusiveness of T1D made it difficult to avoid; thus participants attempted to limit its influence on their lives where possible.

In this study, food constraints were found to be a major factor in participants’ perception of T1D as a negative phenomenon that made them feel different, causing much frustration. Bill, illustrated this when he said:

‘… like kinda hard cause if you have like something nice on the table and you have salad like you have to go for the salad cause don’t want my sugar to get high.’ (male, age 13)

The constraint was largely perceived as negative with a view to limiting adolescents’ choices regarding not only their diet but also meaningful social interactions. Joy expressed it as follows:

‘… it’s hard sometimes when my friends going out to parties and stuff. I can’t drink that or eat that stuff like that, so sometimes it’s hard.’ (female, age 16)

This sentiment often prevented participants from engaging in the normal process of exploring life.

They furthermore regularly engaged in risky food behaviour by consciously eating foods outside of their dietary plans. These experiences were perceived as positive because they allowed them to feel that they were just like everyone else, resulting in moments of normality. In this respect, Frank said:

‘My parents told me not to eat certain foods, my grandparents don’t actually care that I have diabetes, so they go wild every time when I go there to eat whatever I want to. Whatever I haven’t eaten in a long time. I can just. Have fun, basically.’ (male, age 14)

However, it should be noted that friends would often assist the participants to avoid temptation, as has been discussed under the rubric of the first theme above. For example, Frank (male, age 14) indicated that friends would ‘… go get another bowl of cereal and then pour the right amount or they pour it for me.’

#### Subtheme 2: Living with type 1 diabetes leads to a sense of being different

Adolescence is a period marked by attempts to find a place in society, a sense of belonging and a personal identity. However, adolescents with T1D must negotiate its successful management, which often leads them to feel that they are different. These feelings were mitigated through participation in sports. Sport plays an important role in assisting adolescents in finding their role in society, making them feel normal and socially competent.

The participants perceived themselves as being different because of their T1D care plans. This difference was experienced in a variety of contexts amongst which the school environment being the most salient. Frank said:

‘I was the only one in my age group and stuff like that was quite hard to deal with because knowing that nobody else is going to miss the stuff that I am going to and that there was no one I could ask for help and stuff.’ (male, age 14)

Alice expressed herself around this by saying:

‘Like I kinda feel like the odd one out because everybody you know does certain stuff and then if my blood sugar is low then I have to sit you know in a corner …’ (female, age 13)

She continued by indicating the following:

‘Sometimes, um I do sometimes feel left out …’.

These experiences often left participants feeling isolated and left out. Such feelings of difference were exacerbated when participants were teased at school because of the suffering from T1D. Jane said:

‘Lots of people used to make fun of me, they said I was a robot because I had my injections and all that and they thought that was like contagious so that they didn’t want to play with me …’ (female, age 13)

To escape the teasing, Jane (female, age 13) changed schools, relocating to one with a more inclusive environment, because other adolescents with T1D were present there, allowing her to feel more normal. Other participants indicated that they did not tell their peers about their illness out of fear of being teased. Bill said:

‘… I know if I go tell it to everyone they will like go and say things about me, if they get angry with me. So, I don’t tell everyone.’ (male, age 13)

Some of the participants experienced sport as vital not only to managing their T1D but also when it came to fitting in with their peers. They experienced it as a space where they could feel less different and thus engaged in multiple sporting activities. Bill (male, age 13) reinforced this notion when he said: ‘Uh, I do swimming, I do swimming, cricket, soccer, uh and hockey …’. Given the range of activities that Bill listed, there appeared to be multiple attempts to be part of their peer group, which would give them a sense of belonging over and above the issues around their illness. Frank expressed it as follows:

‘Sports is my way out basically. So, playing sport makes me forget about everything else. Makes me feel normal and …’ (male, age 14)

The adolescents also use sport to prove that they were capable of activities similar to those without T1D, and thus normal. Joy said:

‘I usually tell everyone that because I’m a diabetic it doesn’t mean that I’m, I can’t do this, I can’t do that it’s just empowers me to do more. And I’m just like who cares if I have got diabetes …’ (female, age 16)

### Management of diabetes

In terms of the management of diabetes and how it may interact with their illness perception, two subthemes emerged, namely (1) diabetes is challenging and (2) it is often motivated by fear.

#### Subtheme 3: Perception that management of type 1 diabetes is challenging

Participants perceived the management of their illness as challenging because they struggled to cope with the demands entailed with a view to the successful management of T1D. Managing T1D is seen to be difficult because it needs to be done perfectly. Participants felt that they could control only part of it, resulting in not managing T1D. Most participants developed an attitude of just having to live with the illness.

There seemed to be an ambiguity regarding the participants’ experiences of T1D management, where they found it both hard and easy. In a discussion of his experiences around managing T1D, Bill said:

‘Um, my experience has been hard and easy at the same time. So, some days it will be hard but some days it will be easy.’ (male, age 13)

Alice (female, age 13) said: ‘Well it’s kinda difficult … I get really emotional so it’s kinda hard for me …’. The participants further indicated that managing T1D should be easy but remains just hard. Joy said:

‘So I don’t think it’s hard to manage diabetes I think it’s a head it’s a mind game … it’s quite easy, people just make it hard.’ (female, age 16)

This belief that managing T1D is easy but that is hard leads to participants blaming themselves for their failure to control T1D. Frank responded:

‘“I’m not good at taking my stuff and doing it at the right times, so that’s why my sugars aren’t at the place that they should be.’ (male, age 14)

The participants emphasised that they felt pressured to follow diabetes-care plans strictly so as to maintain blood glucose at an optimal level. Bill (male, age 13) indicated that one should: ‘… eat the right things, do the right corrections, always check your sugar.’

Participants felt that their control over T1D was limited, as they could only control part of it themselves. Most of them strictly focussed on control over their food intake and medication adherence. Alice indicated:

‘Um, well there is a little bit of stuff, like I can eat certain stuff and like be on a certain diet.’ (female, age 13)

Jane said:

‘Mm, I think there are a few things you can do but it is very limited because there is only one thing that can bring your sugar down and manage your diabetes, which is insulin.’ (female, age 13)

Because adolescence involves a transitionary stage between childhood and adulthood, it was found that participants generally relied on their family (especially their mother) and friends to assist in controlling T1D; they felt unable to control it themselves or were not ready to do so yet. Jane averred:

‘Yeah, my mom’s probably a really big part of that, cause she comes to all of the appointments and she knows what to do. She probably knows more than me.’ (female, age 13)

Claire revealed:

‘… my best friend, uh she doesn’t want me um to even eat um all the cupcake. She doesn’t even allow me to hold a cupcake for someone.’ (female, age 12)

Frank confirmed the notions arising from these utterances:

‘So, if I take too much spoons [*sugar*] they actually take my food away from me.’ (male, age 14)

The participants often used “we” when discussing the management of T1D. Jane (female, age 13) said: ‘Um, well, we do struggle a bit …’. However, this reliance could lead to additional stress, as illustrated by Alice:

‘Well, I have always worried about like when I get older and then eventually like my parents will like die um and I’m worried about because for my pump we have to put my needle in and my parents always do that because I can’t do it …’ (female, age 13)

Most of the participants held that they valued and depended on support from their family and friends. Despite this help, they still did not feel like it was an easy task, and often felt that they were not managing T1D well. Sue expressed that managing T1D had been challenging:

‘It does become difficult, most of the time I can’t handle it. It’s difficult because then it’s too much to handle for me.’ (female, age 13)

This apparent struggle of dealing with T1D made some participants feel that they were incapable of managing their T1D, and thus they chose not to. Ultimately, they felt as though they did not manage their T1D, as illustrated by Bill (male, age 13): ‘I’m bad with my diabetes so I don’t really manage it’. Participants found it particularly challenging to manage their T1D at school, resulting in failure to do so. Sue indicated the following:

‘Taking insulin at school because most of the time I get busy and something that just holds me up that I can’t take my insulin.’ (female, age 13)

The challenges of managing T1D also came with an enormous amount of pressure, further inhibiting their resolve to manage it. Joy said:

‘… don’t do it because it’s too much work and it’s too much pressure on me.’ (female, age 16)

There was no respite for adolescents with T1D. Because they were overwhelmed by it, they give up on trying to manage it and developed the perception that they just had to live with it – taking on a passive role when it came to managing the illness.

The participants indicated that they just had to live with it and thus had to accept their fate of merely living with T1D. Sue said:

‘I just have to move on and take care of myself.’ (female, age 13)

Bill (male, age 13) added that ‘I just live with it’.

This indicated a resignation to their allotment in life. As John stated:

‘… you’ve got to suck it up, cause it’s your life and it’s not going anywhere.’ (male, age 14)

Joy (female, age 16) reinforced this when she said: ‘But yeah, I like get to live with it …’

#### Subtheme 4: Management of type 1 diabetes is motivated by fear

All the participants experienced fear of future medical complications if they did not manage T1D well. Jane (female, age 13) said: ‘… high blood sugar can be quite worrying, cause I’ve heard of people getting um DKA (diabetic ketoacidosis) and going to hospital and some, some um don’t make it’. This fear, however, served as the main motivation to manage their T1D, given that participants directed their fears to ensuring that they would not experience these medical complications. John expressed this inference when he said:

‘Um, I think when you look at the severity of the long-term effects, I think it kind of is a it’s a motivation to manage it better seeing what it can do to you and what a danger it is. I think if I didn’t know as much I wouldn’t I wouldn’t manage it as well, but knowing the consequences of unmanaged diabetes it helps me manage quite a bit.’ (male, age 14)

Participants imparted that they were constantly reminded of medical complications that would arise if they did not manage their diabetes well, as Bill declared:

‘I didn’t do my sugar that much my mom says like they will cut off your legs you won’t be able to walk you you’ll like your body won’t last longer. So, the like now I’ve started doing my sugar because I wanna live my full life and I don’t wanna get like cut off or something.’ (male, age 13)

Despite this, some participants still felt resigned to merely continuing to experience complications because they felt they were unable to manage their T1D now. Joy expressed this as follows:

‘… I know what’s going to happen in a few years. I feel fine now, but I know what’s coming. So, it’s a scary thing.’ (female, age 16)

And Joy (female, age 16) continued by saying:

‘Yeah, I just don’t want to die before my parents my grans because didn’t listen when I was a teenager.’ (female, age 16)

## Discussion

This study aimed to explore the illness perception amongst adolescents at risk of poor glycaemic control. How this perception contributed to their management of the illness was also explored. Consistent with current literature, negative perceptions of T1D were found to be linked with lower metabolic control.

Adolescents viewed managing T1D as challenging, which confirms the findings of Davidson et al.^36^ that adolescents perceive diabetes as a difficult and demanding condition. According to Hagger et al.,^37^ illness perception may originate from how an individual evaluates their personal experiences of the condition. In the present study, participants’ personal experiences of T1D were not only as challenging as has thus been suggested by these scholars. They moreover struggled to manage their T1D well, exacerbated by the belief that they could only control part of it themselves as well as the immense pressures involved in successful management of the illness. This led to some participants giving up on such management, as they felt that they were not able to manage it perfectly.

Adolescents who perceived enjoying greater control over their condition experienced better adherence to their regimens^24^ and better metabolic control.^38^ Hanna et al.^39^ found that assuming responsibility for self-care is crucial to a successful management of the illness. This is a logical conclusion as, without self-care, the self-management and coping with T1D would be hindered. However, when the participants were unable to meet their management targets, they internalised their failure, resulting in negative self-talk, which affected how they viewed T1D, themselves, and their capabilities. They were resigned to accepting their fate in life, feeling like they just had to ‘live with it’. This finding is in contrast with a study by Jonker et al.^14^ as their participants with well-controlled diabetes perceived acceptance of their fate optimistically. This may suggest that illness perception plays a role in acceptance of the diagnoses of diabetes. Additionally, as adolescents believe that they are invulnerable, they tend to adopt risky behaviours preferring to live in the here and now,^30^ thus further complicating self-management of the chronic condition.

These risky behaviours were predominantly focussed on their strict diets. All the participants discussed the challenges experienced adhering to these. Considering that food consumption is an integral part of socialisation,^40^ which is inhibited by the demands entailed in keeping to a strict diet, the management of T1D becomes necessary. Their dietary restrictions were viewed as negative because they limited their social interactions, making them feel not only different but also like outsiders. This confirms research by Commissariat et al.^41^ who found that adolescents experience T1D and its management negatively with a view to impacting their sense of normalcy and social relations. Furthermore, Marshall et al.^11^ found that adolescents living with T1D perceive themselves as different, culminating in the pursuit of ‘normal’. Pursuing social activities such as sport made the participants not only feel less different but also served as a means to prove that they were just as capable as their ‘normal’ peers, as mentioned.

Despite contrasting evidence regarding support from family, friends and peers in the existing literature, it was found that adolescents rely on the support to manage their T1D. Watkins et al.^42^ found that social support predicted well-being. Additionally, many studies indicate that a lack of parental support was associated with uncontrolled T1D.^4,10,43^ Parental control is, on the other hand, associated with better adherence over time.^24^ However, in this study, most of the participants felt supported by their families, and mothers in particular were found to exert considerable control over management, whilst the adolescents still suffered from uncontrolled versions of T1D. The present study could not determine and evidence reason for this. However, this could be associated with challenges parents experience when they try to strike a balance between holding back and providing support.^44^ This balance is difficult to obtain because adolescence is marked by higher HbA1c levels along with increased demands for autonomy. Many participants indicated that they wanted to take on more responsibilities regarding their T1D management but were anxious about having the necessary skills and knowledge to successfully manage it.

The participants indicated that they had little knowledge and understanding of T1D beyond the medical world. This slant towards medical understanding suggests that the adolescents have not yet internalised or embraced living with T1D, thus tending to not experience T1D as part of their personal identity. Additionally, by refusing to think about T1D and how they perceive it, participants limit their ability to accept living with T1D and perceive it as anything but negative because they only think about it when they face difficulties. These patterns of avoidance and disengagement are associated with poorer metabolic control.^45^ They are also linked with problems in social competency,^45^ which make adolescents who live with T1D vulnerable,^46^ because they may not adhere to their management protocols such as injecting insulin when they are with peers,^47^ nor disclose their diagnosis out of fear for rejection.^48^ The present study confirmed these findings.

As has been indicated, Jonker et al.^14^ found that adolescents with well-controlled diabetes embraced living with T1D and were thus able to have positive illness perception about it, with a successful management thereof. Similarly, Watkins et al.^42^ found that a better understanding of T1D is associated with fewer negative feelings associated with T1D as well as a more positive attitude towards it. A better understanding of T1D is also associated with improved diet adherence.^42^ Adolescents can be empowered to manage their T1D better by providing them with continuous health education from the onset of the condition, whilst limiting threatening messages.

Parental and medical attempts to support adolescents by using threat messages, although motivating at times, do not translate into improved management of T1D. The adolescents believe that because of the difficulties of managing T1D, they would still suffer from T1D-related complications in the future regardless of threats. Ultimately, the threat reminders they receive serve to reinforce their belief that they are incapable of managing their T1D effectively. Lawson et al.^12^ indeed conducted research that indicates that threat messages received are often perceived as negative. Additionally, Edgar et al.^49^ found that the perceived impact of T1D and not the perceived threat to health, nor the management of T1D to prevent complications, predicted more positive management outcomes. Ultimately, the use of threat messages to motivate management in adolescents may have a negative impact on illness perception.^16,49^ This negative perception is linked with higher HbA1c levels.^50^ This is a salient point, because Lawson et al.^20^ found that these perceptions remain consistent over time and that the manner in which health threats are communicated, and indeed how they are perceived, are better predictors of illness perception than personality factors.

The generalisability of this study is limited because of the use of non-random purposive sampling, which limited demographic variability. This demographic variability may limit the scope of the study, but not the transferability of the study. Notwithstanding these limitations, the present study adds to the body of knowledge contained in the literature on adolescents who have found adhering to management protocols challenging.

This study provides new information about the illness perception amongst adolescents with risky control of their T1D in South Africa, as well as insight into how these perceptions affect management. The findings will prompt healthcare professionals to look beyond dietary and medication adherence, thus to include psychological factors such as illness perception when implementing diabetes-care plans and designing interventions. It also lays the foundation for future research around what differentiates well-controlled and uncontrolled T1D and may ultimately lead to developing intervention strategies that will assist people living with T1D to manage it successfully.

## Conclusion

This study demonstrated that adolescents with non-optimal control of T1D assume the belief that it is difficult to manage, which is internalised as personal failure, leading to largely negative perception of the illness. Ultimately, good versus bad management is predicated upon targets that are usually externally imposed.^51^ Adolescents internalise failure to meet these targets as a personal flaw, perpetuating the belief that managing T1D is difficult and not in their control. By focussing on T1D management as predicated strictly on diet and medication adherence, we unfortunately lose sight of the individual. Successful management of T1D relies on many contributing factors and not solely on the adolescent with non-optimal control of T1D.
